# Urinary Diseases and Their Treatment

**Published:** 1839-01-01

**Authors:** 


### Medico-Chirurgical Review,

### No. LIX.

**[No. 19 of a Decennial Series.]**

OCTOBER 1, 1838, TO JANUARY 1, 1839.

I. URINARY DISEASES AND THEIR TREATMENT. By *Robert Willis*, M.D., Licentiate of the Royal College of Physicians, Physician to the Royal Infirmary for Children, &c.

II. TRAITÉ DES MALADIES DES REINS, ÉTUDIÉES EN ELLE-MEMES ET DANS LEURS RAPPORTS AVEC LES MALADIES DES URETRES DE LA VESSIE, DE LA PROSTATE, DE L'URETRE. Par *P. Rayer*, Médecin de l'Hôpital de la Charité, Paris, 1837-38.

OUR readers are aware that at different times we have brought the subject of diseases of the urinary organs before them. Those diseases are important, numerous, and common. They have grown into consequence with the growth of pathology, for this has shewn that complaints referred to other parts had their essential seat in these regions, and that the disorders of the latter were both an index and a result of general constitutional disturbance. This circumstance has contributed to separate these maladies from the confined class of local alterations, and has brought within the range of science what before was the prey of the ignorant and designing quack.

To be "a water-doctor" was formerly a subject of merriment. The astrologer the Katerfelto of the place was the man. In describing Sidrophel, Butler writes—

"To whom all people, far and near,
On deep importances repair;
When geese and pullen are seduc'd,
And sows of sucking-pigs are chows'd;
When murrain reigns in hogs or sheep,
And chickens languish of the pip;
When butter does refuse to come,
And love proves cross and humorsome;
To him with questions, and with urine,
They for discov'ry flock or curing."

But Sidrophel's occupation's gone. Syphilis, the "gravel," the stone, are no longer the no-man's land of physic, but have been investigated, and must henceforth be successfully cultivated, by men of high general attainments. Those attainments have to grapple with no trifling difficulties: for these complaints, perhaps more than any others in surgery, require keen analysis in their discrimination, and the exercise of judgment in their treatment.

Dr. Willis informs us, in his preface, that his object has been to give a comprehensive and connected view of the *Functional Derangements of the Organs which secrete and excrete the Urine*, whether primary or secondary in their nature, *and of the medical and dietetic Treatment adapted to their different Forms.* As among the most frequent and formidable of the immediate consequences of certain disordered states of the renal secretion, he has also included a particular consideration of *Stone in the Kidney and Bladder, and of its Remedy by Medical Means.* He goes on to state:—

"A few years have added largely to our knowledge both of the physiology and pathology of the kidney; and every step in advance, has made the necessity of studying the qualities of the urine in almost every one of the diseases with which the body of man is afflicted, more and more apparent. Under the guidance of science, too, and particularly of recent discoveries in animal chemistry, the examination of the urine, an art but lately held in merited contempt, has assumed a character of the highest importance to the practical physician. If it have been deemed of moment to ascertain the qualities of the alvine evacuations—the mere residue, the undigested and unavailable portions of the food,—it must surely at once be seen that it is of paramount importance to determine the condition of that secretion, which contains the recrementitious matter of the blood, and in its constitution presents an index, as it were, of the state of all the functions whose sum composes the Life. Urinary diseases are besides in themselves, and considered abstractedly, subjects of peculiar interest and importance. They are of very common occurrence, extremely rebellious in their nature, and most fatal in their effects. They are, farther, very far from being generally understood; of all the well-worn pathways of medical literature, there is probably no one less trodden than that which leads to a knowledge of the forms, tendencies, and medical treatment of urinary diseases.

Much has undoubtedly been done in this department of late; but what exists lies widely scattered, is little accessible, and, without connexion, is even less available for practical purposes. I have gathered these fragments together, and striven to arrange them into a whole, under the lights afforded by physiology, reflection, and experience." viii.

These remarks will afford a key to the character and complexion of the work. Like its author it displays the stamp of learning, and will be found to constitute a careful compilation.

The work consists of an introduction and of two parts. The first part is occupied with the consideration of the Functional Derangements of the Kidneys, and their immediate consequences; the second part is devoted to the Functional Derangements of the Organs which excrete the Urine.

The First Part comprises eight chapters. The first is on the morbid states in which the secreting faculty of the kidney is exalted, and the menstruum and readily soluble principles of healthy urine occur in altered absolute, or relative quantity; the second on the morbid states in which the secreting faculty of the kidneys is lessened or abolished; the third, on the morbid states in which the urine contains in excess and as precipitates, certain ingredients that occur normally in smaller quantity and in solution; the fourth, on the morbid states in which the urine contains in solution or as precipitates, certain principles which do not occur in the healthy secretion, but appear to be derived immediately from one or other of these; the fifth, on the morbid states in which certain matters being constituents of the blood are contained in the urine; the sixth, on the morbid states in which the constitutents of secretions and altered elements of the blood are discharged with the urine; the seventh, on the morbid states in which principles foreign to the urine and the blood, and derived from none of the natural constituents of these fluids, are eliminated by the kidney; and the eighth, on the consequences of one or other of the morbid states described, particularly of those comprised in Chapters III. and IV.—Urolithiasis, the formation and growth of urinary calculi.

The Second Part presents five chapters. The first is on impediments to the discharge of the urine; the second, on inability to retain the urine; the third, on irritability of the bladder—Cysterethismus; the fourth, on spasm of the bladder—Cystospasmus; the fifth, on catarrh of the bladder—Cystorrhœa.

The Introduction, consisting of twenty-six pages, is occupied with the physiology of the kidney, and natural constitution of the urine. On these points it will not be necessary for us to dwell long.

Animals, it is well known, are composed of oxygen, hydrogen, carbon, and nitrogen. Their food may or may not contain the latter, but carbon and nitrogen, which, form elements of composition, must be partially got rid of. There is an apparatus, therefore, for throwing off both. A lung, observes Dr. Willis, or something analogous to it, is the apparatus in reference to the carbon; a kidney, that in reference to the nitrogen. The lung and the kidney, consequently, taken together, constitute the apparatus of rejection among animals. It has long been known that no animal could survive continued abstraction from the access of air; that the elimination of carbon, effected by the contact of the atmosphere, was necessary to its existence, and that an organ or means of some kind for effecting this object was as universal as organisation itself. Recent researches have also shown that we descend very low in the scale of creation before we lose traces of a kidney or apparatus for freeing the system of nitrogen. The long, and from their magnitude alone, evidently important Malpighian canals in Insects, have been found to secrete, and even to contain calculi of, uric acid. The same substance has also been discovered in the matter elaborated by the *saccus calcareus*, the *organe de la viscosité* of Cuvier, of Mollusca; and, as in the case of the lung, when we have lost traces of a concentrated and special organ that might be called a kidney, we find evidences of a means diffused through the system for accomplishing the important end of azotic purgation.

The principles excreted by the lung and the kidney represent very nearly the sum of those introduced as aliment, which may be regarded as finally decomposed into carbonic acid and urea.

"The experiments of M. Chossat upon this matter are of the highest interest. He showed that the quantity of solid urinous excrement was in harmony directly with the quantity combined with the quality of the food taken. Each ounce of farinaceous aliment, bread, yielded regularly 9.9 grains, each ounce of albuminous food 13.6 grains, and each ounce of fibrinous food 17.3 grains of solid urinous excrement. They therefore stood to each other in the power to produce solid urine, in the ratio of 5.7.9. When like weights of these different kinds of food deprived of water were taken, the differences in the proportion of urinous excrement yielded by each were still more striking; each ounce of dry bread, for instance, then produced from sixteen to nineteen grains, each ounce of dry albumen seventy-three grains, and each ounce of dry fibrine seventy-six grains of solid urine. Whence it follows that the quantity of azote contained in the food is a principal element determining the amount of solid excrementitious urinary matter. M. Chossat found, in fact, that ten-elevenths of all the azote ingested with the food were discharged by the kidney." xviii.

As Dr. Willis observes, these experiments of M. Chossat's go to place the kidney in the same rank as the lung, and sufficiently explain the serious disturbance of the health that results from impairments of its function.

We need not, at present, examine the anatomical structure of the kidney, and it is scarcely necessary to observe that the performance of its functions depends in a great measure on its nerves. This might have been anticipated à priori, and has received experimental confirmation from Müller and Peipers. When the renal nerves were destroyed or divided, the secretion of urine was entirely suspended.

The azotic compounds in the urine present themselves under the two principal forms of *urea* and *uric acid.* The former has been discovered in the blood; the latter, hitherto, has not. One, or both, with water, are probably the essential constituents of the urine.

The *specific gravity* of the urine varies with the quantity and quality of the food. It varies from 1·005, after a sparing meal with considerable quantities of diluents, to 1·033 or even 1·038, after a hearty meal, with chylification going on.

" Writers differ very much in their estimates of the *average density* of the urine. Prout believed that from 1.010 to 1.015 might be about the average density ; any such estimate from 1.015 to 1.025 is certainly much too high for the latitude of London. My observations would lead me to say that the average specific gravity of the urine among grown individuals generally, could not be estimated more closely than 1.015. If children be included, an estimate somewhere about 1.012 will, I believe, be found very near the truth. Densities below 1.012, I should incline to speak of as *low :* densities above 1.020 I should certainly regard as *high.* But it is next to impossible to come to any conclusion on this point. The density of the urine in fact is influenced by so many varying circumstances—temperature, kind of food, quantity of drink, state of the alvine evacuations, &c. that we are only in a condition to judge of the point in each different individual instance. M. Chossat found the mean density under a vegeto-albuminous regimen in his own person to be 1.012 ; under an albuminous regimen it was 1.015 ; under a vegeto-fibrinous and a wholly fibrinous regimen 1.023 and 1.024." xxvii.

*Solid Matters of the Urine.*—The qualitative analysis of the urine is not difficult—the quantitative is. The analysis of the urine generally quoted is that of Berzelius, as old as the year 1809. It is as follows :—

| | |
|---|---|
| Urea | 30.10 |
| Uric or lithic acid | 1.00 |
| Free lactic acid<br>Lactate of ammonia<br>Extractive matter soluble in alcohol<br>Extractive matter soluble in water | 17.14 |
| Mucus of the bladder | 0.32 |
| Sulphate of potash | 3.70 |
| Sulphate of soda | 3.16 |
| Phosphate of soda | 2.94 |
| Phosphate of ammonia | 1.65 |
| Phosphate of lime and magnesia | 1.00 |
| Chloride of sodium | 4.45 |

Hydrochlorate of ammonia.. .. .. .. .. 1.50

Silicia, or silicic acid .. .. .. .. .. .. 933.00*

Dr. Christison has given a qualitative analysis of the urine of a healthy individual, of specific gravity 1·029, which was voided to the extent of about thirty-five ounces daily. Of this urine the solid contents amounted to 67·7 per 1000. Of these 55·2 were urea, extractive matters, and animalized acetates, soluble in alcohol; 11·1 alkaline muriates, sulphates and phosphates; 1·0 earthy phosphates, and 0·4 mucus. But Dr. Willis judiciously remarks, that this must be regarded as a specimen of urine of unusually high specific gravity. He observes:—

"I have found such differences in the relative quantities of the matters soluble in alcohol and in water, that I believe it to be no more possible to fix on any absolute standard, in regard to the ratio of the animal matters to the salts, in the urine, than to fix on any term as a measure of its specific gravity. This must be apparent when it is seen that in Dr. Christison's analysis, the animal matters are to the salts nearly in the proportion of five to one; in Berzelius's they stand to each other nearly in that of three to one; whilst Dr. Bostock has, if I remember rightly, estimated them as existing in the ratio of about five to four. The result depends greatly on the strength of the alcohol employed." xxviii.

Perhaps some of our readers are not aware that urea, with one or two other proximate animal principles, may be formed artificially.† The mode of obtaining urea is as follows:—When a quantity of cyanate of silver is treated with a solution of hydrochlorate of ammonia in water, a double decomposition takes place; chloride of silver, which is perfectly insoluble, falls to the bottom, a quantity of cyanate of ammonia remains in solution; this is readily obtained in the solid crystalline form by careful evaporation, and in elementary composition agrees exactly with the urea obtained from urine.

At the termination of his account of lithic acid, our author observes:—

"There is one circumstance, which may probably influence the solution of this substance, which has received less attention than it deserves; and which I now find I have myself overlooked in the proper place; it is this: that the combination between the lithic acid and the water must take place at the *moment of nascence*. It is familiarly known that several chemical combinations can only be effected by presenting the combining elements to one another in the nascent state. When the urine contains any lithic acid dissolved, the deposit of this substance from the menstruum is a mere question of time: sooner or later it always happens. The recent researches of Leibig and Woehler have rendered it probable that lithic acid is a compound of urea and a substance itself composed of cyanogen and carbonic oxide, a substance, which, by the action of certain reagents, (superoxide of lead,) and probably also by the deranged operation of the kidney, is converted into oxalic acid, and the peculiar matter found in the allantois of the calf, allantoin." xxxiii.

---

* Dr. Willis's work, for that of a librarian, is carelessly written and got up. This analysis of the urine from Berzelius is a striking instance of the latter;—933 parts of silicic acid! The analysis usually quoted as that of Berzelius, is—

Siliceous earth .. .. .. .. 0.03

Water .. .. .. .. .. .. 933.

† It is on this point that the inverted pyramid of Pantheism, has been poised by some of that metaphysical school.

After a brief survey of the names and labours of those who have contributed to render our information on the morbid states of the urine what it actually is, our author concludes by quoting and recommending for general adoption, Dr. Bostock's plan for noting the characters and qualities of that fluid in disease. We adverted to it in our last number, but it would be well to re-introduce the suggestions of Dr. Bostock here. With some slight modifications and additions, his formula, or proposal includes,—1. The designation of the circumstances under, and the time at which, the specimen of urine considered was voided; 2. Its quantity, and the quantity voided during the preceding twenty-four hours; 3. Its external characters,—colour, odour, transparency, or turbidity; 4. Its specific gravity, and the average specific gravity of the whole of the urine discharged during the previous twenty-four hours; 5. Its state in regard to acidity, neutrality, or alkalescence; 6. The amount of solid contents per cent.; the respective quantities of the extract soluble in strong alcohol, and in water being distinguished; 7. The effects of heat, of nitric acid, before and after the application of heat, of corrosive sublimate, and ferrocyanate of potash; 8. The amount of precipitate thrown down by ammonia, and by oxalate of ammonia; 9. The nature of the deposites, when any; 10. The nature of the spontaneous changes undergone within twelve hours, and of those that take place in the course of two or three days.

With this we terminate our notice of the Introduction, and proceed to the first part of the work, on—

### I.—FUNCTIONAL DERANGEMENTS OF THE KIDNEYS, AND THEIR IMMEDIATE CONSEQUENCES.

### 1.—MORBID STATES IN WHICH THE SECRETING FACULTY OF THE KIDNEYS IS EXALTED, AND THE MENSTRUUM AND READILY SOLUBLE PRINCIPLES OF HEALTHY URINE OCCUR IN ALTERED ABSOLUTE OR RELATIVE QUANTITY.

This chapter is sub-divided into three sections:—the first, on the discharge of urine which is characterized by deficiency of solid matters generally—Hydruria. The second, on the discharge of urine which is characterized by a deficiency of urea—Anazoturia. The third, on the discharge of urine which is characterized by a superabundance of urea—Azoturia.

The reader is already introduced to three of many new names; new, at all events, most probably to him. Whether the advantage of uniformity in nomenclature counterbalances the inconveniences of change of name, is a point which we shall not now pause to discuss. But we are sure that authors ought to be chary how they dabble in the Lexicon for novel terms. What trouble his nomenclature must have cost Mason Good! Yet who knows or cares to know his learned designations for familiar maladies?

#### SECTION I.—*Of the discharge of Urine which is characterized by deficiency of solid matters generally*,—HYDRURIA.

If an individual drinks a great deal, he voids a proportionate quantity of urine. But this is not disease. Yet occasionally there seems an insatiable demand for fluid, in order to supply the kidney which voids it in excess. Dr. Willis has met with the following case, in the course of his reading on the subject.

*Case.* It was that of a small artizan, aged fifty-five, who came into the Hôtel Dieu of Paris, to be treated for a bruise of his knee, from which he speedily recovered. The uncommon thirst of this man, and the incessant calls he had to make water, attracted attention, and it was found that from the age of five years, he had had a constant thirst upon him, and been affected with a diuresis commensurate with his thirst. From the age of sixteen he had not drunk less, on an average, than two bucketfuls of water daily. During the ten days he remained in the Hôtel Dieu, he consumed on an average thirty-three pounds of water every day, often swallowing two litres (above two quarts) at a draught; his solid food was about one pound and three-quarters, and he seems also to have had a little wine. His evacuations daily were about thirty-four pounds of urine, and, at the most, one pound of fæces. Yet this man looked and seemed well; he possessed the ordinary strength of a little man of fifty-five; he was the father of several children, and suffered no inconvenience save from the necessity of drinking, and voiding his urine so frequently. The urine scarcely exceeded pure water in specific gravity. Reduced by evaporation, and having yeast added, it gave no sign of fermentation. The urine was certainly quite healthy, only very much diluted.*

Dr. Willis quotes several other cases of a similar description. They are chiefly obtained from foreign sources, and do not seem to us to be perfectly free from the taint of exaggeration. Shall we receive without misgiving the following case?

*Case.* A woman, aged 40, and the mother of many children, had suffered from continual thirst, and the discharge of a profusion of fetid urine since the time she was a child. Compelled to quit her father's house on account of the quarrelling to which her large consumption of water gave rise, she came to Paris, and entered into service. Here the increased quantity of water that was used in the family attracted attention, and it was found that the servant-girl was in the habit of drinking two or three pailfuls of the pure element every day. A shoemaker having paid his addresses to her, she contrived to conceal her infirmity from him till after the marriage, when of course he discovered the real worth of his bargain, and found that all his earnings were not sufficient, in the depth of Winter, to buy his wife water, so that he had to collect and melt snow and ice from the house-tops for her use. This woman enjoyed very good general health.

To this head of Hyperuresis, are to be referred the copious discharge of limpid watery urine on the conclusion of the hysterical paroxysm, and even the occasional diuresis under which subjects of nervous and excitable temperaments labour. The qualities of the urine, in hysteria, seem to vary considerably. Mr. Cruikshank found it to abound in saline matter, but to

---

* Case reported by M. Boissat, in the Recueil period. de Sedillot, tom. lxxx. p. 164.

contain hardly any urea and colouring matter. Nysten detected a considerable quantity of urea, but little of the peculiar oily extractive matter; the lithic acid and salts he reports as in health.

"My own experiments," says Dr. Willis," would lead me to infer, that in hysterical urine, generally speaking, the characteristic ingredients were not essentially altered in their quality or relative proportions, although they are in very small amount, when their ratio to the menstruum is considered. I have always been able to detect urea; and if any salt has appeared to me more abundant than another, it has been the chloride of sodium. When the urine can scarcely be discovered to differ from spring water, by the areometer, the presence of common salt is still often to be detected by the taste, and I have never seen a solution of the nitrate of silver fail to produce considerable turbidity in hysterical urine even of the lowest specific gravity; but its distinguishing ingredients are always in such small proportion, that its specific gravity is not more than from one to two or three in the thousand higher than pure water." 5.

The urine voided by many nervous subjects in the earlier part of the day is of the same general character as in hysteria. Its specific gravity is sometimes as low as 1·002, yet the urea and alkaline salts may bear to each other nearly the proportions of health. The kidney seems merely to participate in the general irritability of the system. After dinner this subsides, under the influence of the stimulus of the meal; and the patient may now sit for several hours without the slightest inconvenience, and then void ten or twelve ounces of urine at once.

If this be examined, it will often be discovered, although mostly pale-coloured, to be of unusually high specific gravity. Dr. Willis has found it as high as 1·033 at 60° Farenheit, and containing such an abundance of urea, that it yielded a plentiful crop of crystals of nitrate of urea without any preliminay concentration. On standing, this urine almost always deposits the phosphatic salts; in from twelve to twenty-four hours it becomes covered on the surface with an iridescent pellicle; and by and by its reaction is alkaline, although when voided it was acid.

The diuresis under which men advanced in years occasionally labour, is of a similar description. The frequent calls to empty the bladder are accompanied with an augmented flow of urine. Dr. Willis relates a case, but it is defective in an essential point, an examination of the body after death. We cannot say that we have ever seen the diuresis of the old proceed to a fatal termination, without some organic lesion. Yet the case exhibits the leading symptoms, and is interesting as a specimen of their character.

*Case.* The diuresis under which the patient, a gentleman of seventy-two, laboured at the time of his death, had existed for many years, during the three or four last of which he was accustomed to pass regularly between six and eight pints, and often as many as ten and twelve pints of urine in the course of the twenty-four hours. He had always three or four, and sometimes more, calls to make water in the course of the night. The urine was at all times very pale in colour, and quite transparent when passed; nor did it almost ever become turbid in cooling. It had little odour, but what it possessed was of the proper urinous character. It reddened litmus paper, even some time after it had been passed. Its specific grevity varied from 1·006 to 1·012. The solid were, therefore, in very small proportion to the liquid parts; but they were of the usual kind, being made up in nearly equal proportions of urea and extractive matter soluble in alcohol, and of the ordinary earthy and alkaline salts, among which the phosphates greatly predominated. This urine concentrated, and set aside with yeast, showed no symptom of fermentation, and therefore contained no sugar.

The most remarkable constitutional symptoms were languor and disinclination to exercise. The appetite was rather impaired; the thirst was increased; the temper peevish; emaciation inconsiderable.

"There can be no doubt but that such a state of things must hasten the natural decay of the body. The rest broken every hour to satisfy a call to void the bladder were alone sufficient to influence the health most prejudicially. The diuresis of men somewhat advanced beyond the middle period of life is, therefore, a symptom which ought not to be neglected. The disease may indeed go on for years, in some cases, without apparently affecting the general health; but it always tells on the system in the long run; and the prognosis, though it may not be immediately, is never otherwise than remotely, unfavourable." 9.

The only morbid condition of the system with which Dr. Willis can connect this form of diuresis, is the nervous temperament.

*Treatment.*—As no determinate alteration of the urinary organs occasions this form of disorder, the treatment must necessarily be general. The functions of the stomach and alimentary canal are to be carefully attended to. The diet must be nicely regulated, in order that all the food taken may be properly digested and assimilated: and no more fluid should be allowed than is imperatively required to allay feverish sensations of thirst. The greater the quantity of fluid consumed, the more are the kidneys required to free the circulating mass from superfluous water; and organs kept in a state of constant excitement, often end by acquiring a habit of acting in excess. The functions of the skin should be looked to. It is almost always dry and unperspiring. This state must be remedied by the use of the warm bath and flesh-brush, the bath being taken every day or every other day, the brush diligently employed night and morning; and by warm clothing.

"With regard to medicines, these must be for the most part selected from the class of anodynes and tonics; as much of some mild aperient, too, as will secure the due action of the bowels, is indispensable. Probably there is no better compound, under circumstances of the kind we have in view, than a mixture of rhubarb and aloes, in combination with extract of hyoscyamus, which will be found to accomplish more than one of our indications. Mercurials seldom do good in an irritable state of the system like that which accompanies great discharges of watery urine. Their use, even in the way of alteratives, can be very well dispensed with. Narcotics, and of these the chief, opium, are often of signal service in allaying the irritable condition that is proclaimed by hydruresis. To the youthful we prescribe these remedies with reserve; but among the aged we can use them freely. In the case of the old gentleman I have detailed, tincture of opium, even in small doses, had always the effect of lessening the flow of urine, in abating the thirst, in procuring quiet nights, and in increasing the strength; but being a dabbler in physic himself, he never could be prevailed upon to push the medicine far enough to give a decided check to the disease, which I believe might almost at any time have been done, and his life thereby prolonged.

Of tonics, the infusions of bark, gentian, and quassia, in conjunction with the carbonate of iron, will be found the best suited to these cases, and may always be prescribed with an assurance of proving beneficial." 11.

SECTION 2.—*Of the discharge of Urine which is characterized by a deficiency of Urea,*—ANAZOTURIA.

A copious discharge of urine, says Dr. Willis, characterised not merely by a relative but by an absolute deficiency of urea, the peculiarly azotic principle of this fluid, although by no means an uncommon occurrence, has either been overlooked or altogether denied by pathological writers. It is, however, one of the many forms of disordered renal function which has been spoken of under the title of *Diabetes*, or *Diabetes insipidus*. And Dr. W. believes that almost all the cases of diabetes reported as cured have been of this description.

The urine, he proceeds, is passed in great abundance; it is limpid, of a very pale straw-colour, or altogether colourless, and has a very slight, faint odour. When first voided, it shews weak acid reaction or is neutral; it rarely precipitates within twenty-four hours, but cannot be kept for any length of time without undergoing change; it becomes faintly ammoniacal, and covered with a fine creamy pellicle on its surface, which shews the minute pearly-white crystals of the ammonio-magnesian phosphate.

This complaint would appear not unfrequent among the children of the poor.

"I have at this moment three children under my care at the Royal Infirmary for Children, affected with urinary diseases, two of whom present the form of malady to which I have ventured to apply the title of *Anazoturia*. The one of these children is a boy three years and a half old, and has been weakly from his birth. His extremities are emaciated, but he has a large, soft, protuberant belly. He is spiritless, and disinclined to move and play about. His appetite is voracious, and his thirst incessant. The poor little fellow will drink nearly a pint of water at a time if allowed, and is not satisfied with less than about four pints in the course of the day and night. He makes water in considerably greater quantity; which was to have been expected; for he has a constant craving for food, and cries until he is indulged with every thing he sees—meat, bread, potatoes, cabbage, and raw lettuce. The bowels are constipated, and the feces present pieces of undigested aliment, particularly potatoe, carrot, &c. The urine first brought to me by the mother was so free from every one of the sensible properties of urine, that I insisted it must be the rinsings of the bottle, returned into it by mistake; but I soon satisfied myself that I had the veritable urine of the child, by getting him to make water before me. This urine did not differ in appearance from common water; it was quite limpid, absolutely colourless, all but free from odour, and neutral when passed. It became in the slightest possible degree opalescent or milky after standing ten or twelve hours. In specific gravity it corresponded as nearly as possible with distilled water; at first I even thought it lighter, but this was from trying it with a new hydrometer, the scale of which I immediately discovered to be set about a degree too low. Brought to the boiling point it let fall no precipitate, a few bubbles of air (probably carbonic acid gas) being disengaged. No effect followed the addition of a solution of oxalic acid. Caustic potash caused the subsidence of a few flocculi; 1000 grains evaporated slowly left but a fraction of a grain of residue, which appeared to consist entirely of mucus, lithate of ammonia, and the phosphatic salts; but it must also have contained a small quantity of urea and colouring matter; for the urine kept during two days in a temperature of about 65° Farenheit, became very faintly ammoniacal. The quantity of residue I had to deal with was too small to enable me to speak with greater detail." 13.

Dr. Willis refers to several recorded cases or groups of cases, as instances of this affection. But, on analysis, these cases, as he observes, have many features in common. They were all characterized by thirst, gnawing sensations at the pit of the stomach, white furred tongue, constipated bowels, and a parched state of the skin. There was also more or less of emaciation, considerable loss of strength, and generally great depression of spirits in all. The hyperuresis in this class of cases is, therefore, a much more important symptom than in those where it occurs without obvious implication of the functions of nutrition; there is unquestionably disordered assimilation at work here, and the altered nature of the renal secretion is but one indication of a deeply-seated constitutional malady. In children the complaint has appeared to be intimately connected either with the irritation of teething, or a disordered state of the stomach and bowels consequent upon weaning and the use of improper food.

The disease wears an unfavourable aspect. Some of the apparent cures have proved only temporary. The treatment recommended by our author is such as reasoning would suggest.

"It must be directed to the restoration of the functions of the stomach, bowels and skin; to the suppression of the habitual excitement under which the kidney labours, and to the confirmation of the strength generally. These indications plainly point to gentle aperients, and to the use of tonic, diaphoretic, and anodyne medicines. Aloes and rhubarb, the infusions of calumbo, gentian, and quassia, and the compound ipecacuanha powder, are the particular medicines that generally agree best in these cases. The diet should be light and nourishing, and made to consist principally of farinaceous articles and animal food. Slops should be avoided. Toast-water slightly acidulated with the muriatic or nitric acid, but taken in no larger quantity than is found indispensably necessary to allay thirst, will make the best drink." 18.

Section 3.—*Of the discharge of Urine which is characterized by a superabundance of Urea,*—Azoturia.

The urine may at any time, and in any individual, contain more than its usual proportion of saline and peculiar animal ingredients.

But under the heads of *diabetes* or *diabetes insipidus,* there has been described a permanent morbid state of the urine, characterised by a considerable increase of fluid as well as of dissolved urea.

"In this form of malady the quantity of urine secreted is usually large, the fluid is transparent and commonly light-coloured, although it has been seen so dark as to resemble a mixture of porter and water, it exhales a faint but proper urinous odour, and shews acid reaction with litmus paper; its specific gravity is commonly high, varying at different times of the day between 1.018 and 1.035; the specimens of the highest density yield crystals of nitrate of urea on the addition of nitric acid after a few hours rest, without the assistance of concentration; the others require a certain though still a small relative amount of concentration before they can be made to furnish crystals of this substance. The density of urine, that might be styled ureous, however, would not appear to be necessarily great. There is, for instance, an 'analysis of a specimen of anomalous urine' in the 19th volume of the Medical Gazette, by Mr. Rees, of which the specific gravity was only 1.008; nevertheless, the amount of urea in relation to the other solid ingredients appears to have been large, for of the fifteen grains of solid extract yielded by 1000 grains of this urine, 10.2 grains were urea. The disease here was obviously a variety of the hydruria." 19.

There are frequent calls to make water, urgent when they happen. The general symptoms are loss of strength, feelings of languor, a greater or less degree of emaciation, &c. The emaciation and debility, however, do not usually shew themselves in a very marked degree, at least in the earlier stages of this disease. Some degree of thirst is very constantly present, and there is often a sense of sinking, and complaints of an uneasy gnawing pain at the pit of the stomach. There is often an anxious, hollow-eyed expression of countenance. The patients are generally of a spare habit, and a nervous temperament, and have always been more or less troubled with a frequent desire to make water. Men who had lived freely in former years, who had been in the habit at one time of indulging in spirits, wine, and other fermented liquors, and especially men who had abused their sexual propensity, have been observed to be the usual subjects of this form of complaint. Yet temperance does not, nor is it likely that it would, secure an entire immunity from its approach. Intemperance is but one cause of morbid action. Unfortunately there are many others. Children are often affected, though in them the disorder is too apt to be overlooked.

"Ureous urine has been observed to alternate with other morbid states of the same fluid, with the albuminous and saccharine particularly, and also with that in which the phosphatic salts are copiously elaborated. I believe it to be the common precursor, as recent inquiries have shown it to be the general attendant of the mellitic diabetes, as well as the state into which that formidable disease passes under the influence of treatment of a certain kind. A ureous state of the urine has also been found to accompany several forms of acute disease. Thus the urine of a young man labouring under peritonitis was found on analysis to contain one-third more urea than healthy urine. In certain continued fevers, too, of bad type, the urea often becomes disproportionately abundant in the small quantity of urine then discharged, and it is under such circumstances that the kidney seems occasionally to separate the elements of urea under other and very remarkable forms, namely, carbonate of ammonia and hydrocyanic acid, which I shall mention by and by in two of the sections of my fourth chapter.

Instead of being absent in the urine of patients affected with the honey diabetes, as was long supposed to be the case, urea has, with the progress of chemistry, been demonstrated to be present, first in a *certain proportion* (Dr. Henry), then in *as large a proportion as in health,* (Mr. Kane), and finally, under certain circumstances, in *considerably larger proportion* than in health (M'Gregor). Mr. M'Gregor, for example, found a patient labouring under diabetes mellitus, who had undergone no treatment, passing 1013 grains of urea daily, the quantity emitted by a person in health being but 428 grains; a second patient was found to be passing 945, and a third 810 grains of urea every day. In a patient fed on beef and water exclusively for three successive days, as many as 43 grains of urea were obtained from each thousand grains of urine; the same quantity of healthy urine rarely contains so much as 30 grains." 23.

Allied as it is to diabetes mellitus, this complaint yet differs from it. First, there is no sugar—no new matter—secondly, there is not the same amount of thirst, of voracity of appetite, of dryness of skin—thirdly, it is more curable.

In a case which occurred to Dr. Elliotson, and which was thought to be diabetes mellitus in the first instance; the urine from 16 pints in the 24 hours, was reduced in the course of a fortnight to two pints, mainly through the effect of opium, and ultimately the patient got well, though he continued subject to occasional relapses. In one of Dr. Prout's cases, though the patient's general health was greatly improved under a course of constitutional treatment, this seemed to have very little influence on the peculiar malady; but a bitter infusion with the addition of potash and tincture of opium, had an immediate effect, and controlled the inordinate and abnormal action of the kidney in a very remarkable manner, so that this patient also recovered, though like the former, he remained subject to relapses, which, however, always yielded to a repetition of the old remedies. A female, aged fifty, had laboured for some time under a diuresis, so that she passed as many as 10 pints of urine in 24 hours. The urine was of a pale colour, and nearly transparent; it had a faintish odour, reddened litmus, and was of the specific gravity of 1.034. The residuum left after evaporation did not seem to differ from that of healthy urine, but it was greatly more abundant, for it amounted to about 9½ ounces daily, which is fully 8¼ ounces more than in health. This patient recovered completely under the use of chalybeates. Dr. Willis's case did well under the use of an infusion of quassia, and a pill containing equal parts of the compound rhubarb pill and the compound powder of ipecacuanha, five grains of each being taken twice a day. In three weeks, his urine was in smaller quantity than it had been for years, and his health, &c. had undergone a proportionate improvement.

If taken in time, then, the ureous diathesis is far from irremediable. "The system," observes our author, "will even be found to bear bleeding, both generally and locally. When any thing like uneasiness in the lumbar region is complained of, cupping from the loins should not be neglected; and this, succeeded by blistering, bitter tonics, chalybeates and opiates, in conjunction with a regulated diet and attention to the state of the bowels and skin, seems adequate to control the disease, even after it has existed for some considerable time unnoticed, or with its actual nature overlooked."

We must add, for the benefit of our readers, the directions of our author for the investigation of the physical and chemical varieties of morbid urine.

"The general appearance, degree of transparency, disposition to deposit, odour, &c. are to be noted. The specific gravity is then to be ascertained, a piece of knowledge easily procured by means of an instrument,—hydrometer or urinometer, sold in the shops for the purpose.* A given quantity of the urine, say 1000 grains, is then to be slowly evaporated, at a temperature not exceeding 160 or 200 degrees of Farenheit, till it ceases to lose weight. The quantity of extract being ascertained by weight, the proportion of the solid matters to the water will become exactly known. The extract is next to be digested with strong boiling alcolol, (sp. gr. .833) which will take up the urea and salts (the lactates) which are soluble in alcohol. What is dissolved is to be poured off; what remains is to be washed once or twice with a little fresh boiling alcohol, which is to be added to the first. The alcoholic solution is then to be reduced by slow evaporation to the consistence of extract, or till it ceases to lose weight, and its quantity ascertained. The saline mass which was insoluble in alcohol, is to be treated with distilled water at 60 degrees Farenheit, two or three times, and the different solutions being added together and evaporated to dryness, the quantity of saline ingredients,—the soluble chlorides, and alkaline phosphates and sulphates, will be discovered. The insoluble matters, consisting principally

---

* "Those I have used I had of Elliott, 112, High Holborn. The most convenient apparatus for evaporating urine with which I am acquainted is Dr. Arnott's ingenious and admirable stove. I have reduced many pounds of urine by its means, the fluid never having exceeded the temperature of 100 degrees Farenheit."

of the earthy phosphates which remain on the filter, being dried, are to be weighed. They are afterwards to be digested with caustic potash; and being dried and weighed again, the quantity of mucus or other animal matter will be estimated by the loss of weight, if any, which is sustained. This is as much as is required for medical purposes. For every information requisite in conducting a more minute analysis of the urine, I beg to refer to the excellent treatise of Mr. G. Rees, entitled, 'On the Analysis of the Blood and Urine in Health and Disease.' 8vo. London, 1836." 27.

### II.—MORBID STATES IN WHICH THE SECRETING FACULTY OF THE KIDNEY IS LESSENED OR ABOLISHED.—ANURIA (ISCHURIA RENALIS, CULLEN; PARURIA INOPS, GOOD).

Dr. Willis prefers the term anuria to that of ischuria renalis. The latter implies an impeded discharge of urine, whereas the affection is diminution or suspension of the secreting power of the kidney.

We have a familiar instance of diminution of the urinary secretion in acute diseases. It is scanty, high-coloured, and loaded with salts. After severe surgical operations, too, the secretion of urine is often greatly affected. When the suppression continues for any length of time, the case will almost certainly end unfavourably. The anasarca that succeeds scarlet fever probably depends mainly and immediately on deranged function of the kidney. The urine is generally all but suppressed in the earlier stages of the attack, and the albuminous state of the fluid gives strong assurance that such is actually the case.

" A condition of the renal function, and of the urine elaborated, having precisely the characters above enumerated, but without the local mischief, or preceding inflammatory fever to account for it, is a circumstance which is occasionally observed. This occurs most frequently among young children, and has been well described by a pathological writer of high repute on the Continent,* under the title of Urodialysis neonatorum (Harnscharfe der Kinder). I have myself met with the complaint oftener than once. Infants affected in this way, void very small quantities, often only a few drops at a time, of extremely high-coloured urine, which stains the linen of a deep reddish yellow. It seems to be passed with great pain, and very much as if it were molten metal, the little patients drawing their legs up to their belly, and crying bitterly as it comes away, and it very obviously scalds the surfaces over which it passes, and excites inflammation in the mucous lining of the bladder and urethra, as appears by the increased quantity of mucus which before long begins to be excreted along with the urine. With such a state of things, there is, of course, more or less of febrile excitement associated. The skin is hot and dry, the thirst is increased, and the bowels are obstinately constipated; the feces are only voided in the shape of little rounded masses like marbles; the digestion, too, is obviously impaired, and the breath smells strongly of vinegar. As in so many other urinary affections, the skin in this one is generally irritable, and the seat of eruptions of different kinds. It is always kept whole with great difficulty; wherever two surfaces are in contact, they almost certainly inflame, and by and by pour out a thin, sharp,

* " Professor Schoenlein, Allgemeine Pathologie u. Therapie b. iii. s. 232, also by Jahn, Verwandschaft der Greises-und Kinderkrankheiten, in Hecker's Annalen, b. xii. s. 129."

fetid fluid, which causes the mischief to spread. Sometimes the cutaneous affection assumes a more determinate form, and appears as psydracious pustules scattered over different parts of the body; these break, and give rise to superficial and often very troublesome sores, especially in the groins, axillæ, folds of the neck, &c." 30.

An analogous affection is occasionally observed among persons in the decline of life. Our author christens it (the term is imported from the German) urodialysis senum. The hard names thicken on us. Were the same plan applied to the diseases of all the other organs we should have a very pretty vocabulary. This method has been used in the diseases of the eye. With them, indeed, it is indispensable, the affections being so numerous and the shades of difference so slight, that arbitrary symbols are more convenient than diffuse general expressions. Yet who, even in the instance of the eye, has not felt that the names were as hard to remember as the things? We greatly doubt the advantage of putting the kidney again in the baptismal fount, and standing god-father to its morbid brood.

Be that as it may the "urodialysis senum" appears to us to be a newer name than thing. It is like plain John in masquerade, with a fine title and a large nose. For, setting aside some accompaniments of a bad appetite, acid eructations, and torpid bowels, we find it either an attack of gravel, or a threatening of gout, such as our "fine old English gentlemen" have had from time immemorial, and all of us have treated without thinking of erecting it into a new disease. However, Dr. Willis gives it yet another name—*anuria pyretica*, to distinguish it from ischuria renalis, which he thinks would be better designated *anuria apyretica*.

His account of anuria or renal ischury is precise. It generally comes in the midst of apparent health. It is most frequent in the young and old. In the adult, the complaint begins with malaise, occasionally some degree of pain in the loins, or flank, or whole of the abdomen—nausea, which goes on to troublesome vomiting. There is at the same time very generally associated a singular degree of torpor both of mind and body, marked by disinclination to motion and to occupation of any kind—the patient has mostly a dull and abstracted look, unless when immediately engaged by those about him. When questioned as to his state, he says he feels very well; and it is generally only when particularly interrogated that he remembers it must be very many hours since he passed any urine. The pubic region is examined, and is found without fulness or pain. To have assurance that the bladder is not distended, he is requested to make water; after some delay, he probably passes a spoonful or two, probably not a drop. A catheter is introduced, and a small quantity, or none, is drawn off. Drowsiness may now set in, and slight incoherence may end in coma. Convulsions follow, and death ensues.

Dr. Willis observes that the progress of the disease, and the intensity of the symptoms, vary considerably in different instances. In the generality of cases, coma occurs about the fourth or fifth day from the time when the secretion of urine is totally suspended; and the fatal termination usually happens after the lapse of a few days more. But the secretion of small quantities of urine, which takes place from time to time in some cases, seems to relieve the system in some sort, and then the disease may go on for a much longer period than it ever does when the function of the kidney is completely annihilated. Under these circumstances anuria may only prove fatal after an interval of several weeks; or, time being allowed for the employment of the proper remedies, it may finally be brought to a favourable issue. There is a remarkable case related by Dr. Laing, of Fochabers, in which not a drop of urine was secreted for between nine and ten days at least, yet in which the patient ultimately recovered completely. But cases far more marvellous have been related. Dr. Richardson described, in the Philosophical Transactions for 1713, the case of a young man seventeen years of age, who had never made water in his life. The Doctor must be supposed to have been an egregious gull.

Our author offers an explanation of these marvellous cases. They are not worth the trouble. There was fallacy or trick somewhere, and, as in the case of animal magnetism, a man of sound sense will think even the investigation of the humbug thrown away. Le jeu ne vaut pas la chandelle.

We quite agree with our author in the following observations on vicarious discharges.

"With the explanations of such cases that have been grounded on the assumption of vicarious discharges of urine by other organs, I feel free to own my total dissatisfaction. I do not believe that any one organ in the human body can discharge the office of any other. The secretions of particular organs being retained, indeed, the whole body, solids as well as fluids, will show traces of their presence. The duct of the gall-bladder being obstructed, the roots of the nails and whites of the eyes soon become tinged with bile, and the blood and the urine yield its peculiar principles to chemical reagents. The urine not being discharged, the mucus of the mouth and the perspiration may acquire a urinous taste and odour. But that the axilla, umbilicus, mamma, external ear or stomach, ever took on themselves the function of the kidney, and secreted ounces, pints, or quarts of urine, I conceive never to have happened, and hold such an occurrence by the fiat of the Creator in establishing a particular organ for the secretion of urine to be impossible. Since general physics and the laws that regulate the functions of organized beings have been more widely and more carefully studied, extraordinary and miraculous occurrences have greatly diminished in number, and such as do now present themselves at distant intervals are very commonly discovered to be deception. The gentry who pretend to perceive colours by the sense of touch, who live for years without taking food, who pass no fæces and make no urine, are all found out sooner or later to be impostors, and those who believed them to have been of easy faith. When we read, therefore, of the discharge of quantities of unmixed urine from the umbilicus, ear, mouth, &c. we naturally and safely discredit the facts, however respectable the testimony upon which they are announced,—Mrs. Mary Tofts gave birth to innumerable rabbits, if the testimony of one of the very respectable members of the medical profession of his day be but taken. I have heard of a poor girl who habitually vomited fæces well formed, and moulded by the cells of the colon; but she swallowed them previously." 38.

We have again and again protested that if testimony is to be trusted, in opposition to reason, there is no lie be it ever so gross, no absurdity be it ever so palpable, that we ought not to believe implicitly. The analysis of evidence is not gone into by the credulous or the knavish, who retail stories, and *cannot* be conducted by the sceptical who get them at second-hand. It is always safest to doubt what is contrary to experience. If the thing is true, its truth will sooner or later be established, and a little delay is the only or chief evil. But if it is false and we believe it, the mischief that is done is inconceivable.

It is often difficult to assign a cause for the disease. Cold, perhaps, is the most common—violence may give rise to it—so may calculi in the kidney, or in the ureters.

Dr. Willis quotes some cases from authors, but we need not introduce them.

The morbid appearances, he says, discovered in the bodies of those who have died of anuria, have been of different kinds. Generally speaking, the kidneys have been found with the appearances of inflammation, and redder than natural; sometimes their substance has been drier and much firmer than it is in a state of health. In some cases one kidney has been observed to be much enlarged, the other being reduced in size, and either of cartilaginous hardness, or preternaturally vascular. Occasionally these organs seem to have been less the seat of active disease themselves, than the adipose and cellular substance amidst which they lie imbedded.

Occasionally, he goes on to observe, the morbid changes exhibited by the kidneys, appear to have been the effect of the mechanical irritation of calculi, or of gravel filling their pelves, as the antecedent symptoms of the disease were undoubtedly caused by the impediment to renal secretion offered by these foreign substances.

"The liver has been found inflamed and partially softened in some cases of anuria; and it is worthy of observation, that instances of jaundice occasionally present themselves, which terminate unexpectedly in coma, without any great urgency of symptoms, just as we see matters come to pass in anuria. Effusion into the ventricles, and upon the surface of the brain, is another morbid appearance often found in connexion with suppression of the functions of the kidney. The fluid in these cases sometimes had a distinct urinous odour. The effusion in adults would appear generally to be a consequence of the interrupted renal function. Among children, on the other hand, the cerebral symptoms commonly precede the suspended secretion of urine, so that the anuria is but one in the train of symptoms which accompany the formidable disease entitled Acute Hydrocephalus." 48.

It is, perhaps, unnecessary to say more respecting the pathology of the disease than that it is exceedingly obscure.

The treatment is necessarily empirical. We must endeavour, as far as possible, to ascertain the cause of the attack. General bleeding, and the local abstraction of blood by cupping, are serviceable. A succession of large blisters on the loins appears to be positively useful, and the Spanish fly has been recommended internally. Purgatives and diaphoretics with saline diuretics, and infusion of digitalis, diluents, and the warm-bath, constitute the stock of remedies in common use, and advised by Dr. Willis.

"Another, and, in some sort, specific plan of treatment has, however, been recommended by very competent authority—Dr. Elliotson.* This consists in the exhibition of the turpentines, and more acrid diuretics, especially cantharides. Cantharides have, undoubtedly, a very powerful and peculiar effect on the kidneys; and I am not prepared to say that some cases of anuria may not have done well under the use of this active medicine; but as the able practical physician, just mentioned, does not say that he had seen any of the cases upon which his recommendation of cantharides as a specific is grounded, we

* Lectures reported in Med. Gazette.

may be allowed to doubt whether the recoveries which took place were a consequence of its administration.

When we have any clue to suspect the presence of gravel or calculi in the pelves of the kidneys, or impacted in the course of the ureters, as the cause of the disease, which I have imagined might sometimes be derived from feelings of distention or swelling, such as were complained of in the first period of Dr. Brown's case, it would be proper to associate with the means already recommended, copious dilution with fluids holding the bicarbonate of potash or soda in solution, such fluids being calculated to act on the lithic acid of which renal and ureteric calculi so commonly consist. The vapour and hot air bath would almost certainly be found powerful auxiliaries under the same circumstances. Emetics also, which have been strongly recommended in the treatment of anuria generally, might be prescribed with an especial prospect of proving useful in that form of it, to which peculiar reference is now made." 51.

We proceed to the Third Chapter, on the—

### III.—MORBID STATES, IN WHICH THE URINE CONTAINS IN EXCESS, AND AS PRECIPITATES CERTAIN INGREDIENTS THAT OCCUR NORMALLY IN SMALLER QUANTITY, AND IN SOLUTION.

Dr. Willis considers sedimentary urine under two distinct heads: first, that in which the lithic acid and the lithates, especially the lithate of ammonia, form the precipitate; and second, that in which the phosphatic salts, namely, the ammonio-magnesian phosphate, and the phosphate of lime, compose the deposite.

### SECTION 1.—*Of the Sedimentary Urine in which the deposit consists of the Lithic Acid and the Lithates,*—LITHURIA.

We need not particularize the characters of the urine which contains lithic acid and the lithates. As it cools it throws down the latter. Dr. Willis observes that—

When collected by decanting the supernatant urine, and dried, the lithic acid thus deposited appears in the guise of a parcel of brilliant crystals, which seem to be four-sided prisms, very like sea-sand or muscovado sugar in appearance, but drier and more gritty, of a brownish-red colour of various shades of intensity, being in some cases as dark as wainscot or mahogany, in others of a light brownish-yellow, and in some rarer cases nearly white. These crystals are very little soluble in water. Dr. Prout failed to dissolve one part of lithic acid in 10,000 parts of cold water. Dr. Henry, on the contrary, states that one part is soluble in 1,720 parts of water at 60° Fahrenheit, a discrepancy which Dr. T. Thompson* very happily explains by supposing Dr. Henry's statement to express the *quantity* of lithic acid which water can dissolve when the attraction of aggregation is destroyed;—Dr. Prout's the *power* which water possesses to destroy this attraction of aggregation. At all events, lithic acid is a very insoluble substance, so much so, indeed, that it has frequently been made a question among chemists to explain by what means it was held dissolved in urine in the quantity in which it frequently occurs in that fluid,

* Art. Calculus, Cycl. of Pr. Med.

Various explanations of this circumstance have been hazarded. Proust was the first who maintained that the lithic acid of the urine existed in combination with ammonia, and was made soluble in the fluid from this circumstance. This explanation has been adopted by Dr. Prout, who denies, in toto, the existence of free lithic acid in the urine. According to Prout, the lithic acid is always present in combination with ammonia, and occurs as a super-lithate.

"But the simple fact that lithic acid in combination only with a little colouring matter, is constantly deposited from the urine, seems of itself a sufficient answer to this view; and farther it happens, unfortunately for its stability, that the addition of a solution of caustic ammonia to urine, is followed, in the first place, by the precipitation of the phosphates, and after the lapse of a few hours by that of the lithic acid also so completely, that an acid subsequently dropped into the filtered fluid causes no further deposite of this substance. Wetzlar, who particularly insisted on the above facts, in order to get over the difficulty presented by the insolubility of the lithic acid, himself maintained, that it existed in the urine in combination with soda. But lithate of soda is itself so extremely insoluble a salt, that the matter is not much mended by the supposition; besides, later researches in chemistry will not suffer it to be entertained, although it is interesting to know that a solution of lithate of soda will bear a considerable dose of lactic acid, the free acid of healthy urine according to the analysis of Berzelius, without undergoing decomposition. By others, the lithic acid has been held to be rendered more soluble by the presence of the saline bodies which occur along with it as constituents of the urine. But direct experiment is against this notion. I have myself added the purified saline ingredients obtained from 1000 grains of urine, to the like, and even to half the quantity of distilled water, without finding that this solution had any greater power of dissolving lithic acid, or of holding it dissolved, than distilled water itself. Dr. Duvernoy, of Stuttgardt, appears, however, to have solved this difficult question at last. He has shown, as the writer thinks in a very satisfactory manner, that it is upon the presence of the peculiar odoriferous colouring principle of the urine that the solubility of the lithic acid, and even of the superlithate of ammonia, mainly depends. This inquirer dissolved two grains of lithate of soda in between two and three ounces of hot distilled water. The addition of a few drops of an acid of more powerful affinity instantly caused the precipitation of the lithic acid in small crystalline plates. But on dissolving the same quantity of the salt in a like measure of fresh urine, Dr. Duvernoy found that he could add the stronger acid till the mixture reacted powerfully on litmus paper, &c., without the slightest immediate precipitation of the lithic acid taking place. It was only after the lapse of several hours that the fluid began to grow turbid, and the lithic acid to be deposited in the shape of crystals and impalpable powder. Again, he found that if to a saturated solution of lithic acid in boiling water so much of the proper colouring matter of the urine be added as will give to the fluid the hue of somewhat concentrated urine, the acid, instead of being precipitated as the mixture cools, will continue in solution long after it has become quite cold. Neither does the addition of a stronger acid to this compound fluid, even when cold, cause any immediate deposite of lithic acid; this only takes place after an interval of many hours." 57.

Dr. Willis presents a Table of the Urinary Sediments, compiled from the observations of Dr. Prout and of Messrs. Vigla and Quevenne.* We subjoin the Table in question.

---

* Etudes Microscopiques de l'Urine; l'Experience, tom. i. Janvier, 1838.

| | |
|---|---|
| Red crystalline sediment, composed of | Lithic acid and the colouring matter of the urine. The crystals are rhomboidal prisms, which in the field of the microscope, appear under the form of pretty regular lozenges, generally of a beautiful topaz-yellow colour singly. Vigla and Quevenne. |
| Lateritious, red, or reddish-brown Sediment, composed of | Lithate of ammonia; purpurate of ammonia and soda; colouring matter of the urine; and occasionally an admixture of the earthy phosphates. Prout. Lithic acid combined with the colouring matter of the urine. Vigla. |
| Yellowish Sediment, composed of | Essentially of lithate of ammonia; a little lithate of soda, colouring matter of the urine, more or less of the earthy phosphates. Prout. Lithic acid combined with less of the colouring matter of the urine, than in the preceding form of deposite. Vigla. |
| Pink Sediment, composed of | Lithate of ammonia; purpurate of ammonia. Prout. Almost wholly of lithic acid; some lithate of soda; animal matter, and a little phosphate of lime. Quevenne cum Vigla. |

It is well known that deposites of lithic acid and its salts take place on the termination of febrile paroxysms. Dr. Willis seems to attach more faith than we think they merit to the critical character of these deposites. No doubt they often are critical, but no doubt they frequently are not. We have again and again seen copious deposites of the lithates in cases of suppurative fever, succeeded, not by recovery, but by death.

Dr. Willis passes from the lithic acid diathesis, to the disposition to deposit the lithic acid and its salts as a special disease, and independently of any febrile disorder in the system.

The periods of life at which this diathesis more especially obtains are infancy, and the years between forty and sixty. Every circumstance, particularly at these periods, which tends to occasion an excess in the acid and saline ingredients of the urine must tend also to cause their separation from the state of solution in which they ought to exist. For the lithic acid is nearly insoluble; its salts are very sparingly soluble, even the lithate of ammonia requiring something like 480 parts of water for its solution. Thus, observes our author, we have but to suppose the quantity of lithate of ammonia in the urine to be accidentally tripled, which often occurs, to have of necessity a portion of it thrown down as a solid, the watery menstruum of the urine not being then in quantity sufficient to preserve the whole of the salt in solution. And then it happens that the affinity of the lithic acid for the bases with which it occurs combined, by which its solubility is somewhat increased, is so slight, that even the weakest of the acids, the carbonic, citric, acetic, &c. are powerful enough to dispossess it, and effect its precipitation in a concrete form.

Of all the causes that engender lithic acid none are more powerful than excess of animal and other nutritious food; and of all the causes that effect the separation of the lithic acid, when engendered, none is so powerful as the presence of a free acid in the body. These facts, which we mainly owe to the labours of Dr. Prout, or rather which became more generally known in consequence of those labours, have the most direct and important bearing upon practice.

Dr. Willis observes :—

"Do we not in fact see the infant living on a highly azotised and peculiarly animal food, which has further a singular tendency to acescency, precisely the two conditions requisite to the production and precipitation of lithic acid? Can we wonder, then, that urine which deposits lithic acid and its salts, should be common in infancy? especially when we add that the children of the poorer classes who are most obnoxious to this state of urine, independently of their immensely larger relative numbers, are peculiarly open to all the other influences that are held favourable to the inducement of deposition from the urine—such as exposure to cold in consequence of scanty clothing and indifferent lodging, and to derangements of the stomach and bowels from coarse and improper food.

Again, when we regard the age between forty and fifty, the next period in human life that is marked by liability to the precipitation of crystalline lithic acid from the urine, and cast an eye upon the men constituting that larger portion of the community who by manual labour earn their bread in the sweat of their brow, do we not see the influence of incessant toil beginning to be felt by the system about this time? Exposure to wet and cold has now withered the skin, coarse food has impaired the digestive powers, hard labour has blunted the sensibilities, and the frame, which but a few years before seemed knit to endure for an indefinite period, already shows symptoms of decay. Or, turning to another large class in society, composed of the men who by the exercise of their intellect are girt for the race of life, do we not see the struggle very generally concluded about or soon after the age of forty? It is now that men in professions and general business either discover that they have been using undue exertion, and have suffered in body, or that they first begin to surround themselves with something more than the bare necessaries of existence. The simple meal, hitherto hastily snatched at some lull in the storm of business, now no longer suffices; arduous labour both of mind and body craves relaxation, and long abstinence deserves indulgence. It is now that a man begins to dine in earnest, to entertain his friends, and in his turn to be entertained by them. The stomach is taxed to the performance of unusual duty, and not uncommonly taxed beyond its powers; the blood is fevered by strong drinks of every kind; the nervous system is excited by the presence of company, &c. The pabulum of lithic acid is furnished to the kidney in abundance, febrile excitement leads this organ to exert its peculiar acidifying powers upon the matters presented to it, and imperfect digestion supplies an acid—all that was wanted to bring about the deposition of stone-ware, which now so frequently occurs. Happy if this take place without the body, and not within the pelvis of the kidney!" 63.

The facts embellished by the extraneous ornaments of Dr. Willis have been long known, fully laid down in lectures and in books, and form the canons of belief and the principles of action to the best practical surgeons and physicians. But we apprehend that Dr. Willis has either slightly mis-stated the question, or else that it is generally misunderstood. It is commonly believed that the subjects of stone, or in other words of the lithic diathesis, are the very young and those advanced in life. But it is also believed that it is the young of the poorer classes and the old of the upper classes. We never conceived that *infants* were particularly prone to this disorder. If the children of the poor then are the subjects of the complaint it is not because their food is highly azotised. It is not during the period of suckling that they suffer, it is when they begin to live on bad and innutritious food, when their digestive organs are disturbed by want of nutriment and by unwholesome nutriment. If highly azotised food in infancy, or even in early life, gave birth to the lithic diathesis and stone, the children of the rich ought to suffer most. All practical men will agree, we apprehend, in stating that they do not.

Luxurious living is, no doubt, a predominant cause of the prevalence of the disease after middle-life in the upper classes. In the lower, the same age displays a similar liability, though not to a similar extent. We conceive that, in this class, and indeed in both, the tendency to organic diseases of the kidney, after the vigor of life is gone, has been too much disregarded in estimating the production of urinary calculi. It is this tendency, which especially renders operations on the old so much more fatal than operations on the young. The concomitant alterations of other organs contribute, of course, to the same effect.

Dr. Willis goes on to remark :—

"Nor is indulgence in large quantities of rich food and strong drinks efficient in promoting a tendency to the deposition of lithic acid merely from supplying the kidney in abundance with the materials for its formation. They are further influential in this direction from causing a positive decrease in the quantity of urine secreted. Not only are the saline and acid ingredients of the urine increased in quantity, but the menstruum in which they are carried forth of the system is diminished in amount. In infancy the deposition of lithic acid would unquestionably be more frequent than it is, were it not for the state of copious dilution in which food is then taken. And it happens fortunately, perhaps, that the unnecessarily large quantities of highly nourishing food in which grown men are so prone to indulge, are mostly associated with pretty copious potations of different kinds ; good eating craves good drinking, and wine, beer, and tea and coffee, are always swallowed abundantly to allay the fever which heavy meals of highly-seasoned animal food never fail to engender. Still the urinary secretion even under these circumstances is rarely found to be regularly in the ratio of the liquid imbibed. Animal food and strong drink seem to have a positive repressing influence on the secreting functions of the kidney. Very little urine is passed for twelve or eighteen hours after a debauch in meat rather than in drink. During all this time the function of the kidney is nearly in abeyance, and the salts of the urine are separated *suspended* rather than *dissolved* in that fluid. It has been remarked, too, that carnivorous animals, though they drink water freely, make but very little urine in comparison with the herbivorous tribes, many of which, living on succulent vegetables, rarely drink at all, and yet pass large quantities of urine. A knowledge of this fact led Dr. Wollaston long ago to propose that persons liable to gravel should follow a vegetable regimen; and Dr. Pearson afterwards remarked that the calculous concretions of phytivorous animals never contained lithic acid, and followed Wollaston in recommending vegetable diet. The peasantry of Scotland and Ireland who live almost entirely on vegetable matters, often of the coarsest kind, and drink nothing but water, actually labour under a kind of diuresis in contrast with their brethren of England, who live on bacon and cheese, and wheaten bread, and drink little but ale or beer ; and lithic urine and its consequences are rare out of the towns both of Scotland and Ireland." 65.

When we reflect on the quantity of water that enters into the composition of the fresh vegetables on which the herbivora live, we cannot be surprised at their urinary secretion being copious. Nor can we be astonished at animal food, which contains so much less water of composition, not occasioning the same amount of that secretion. Yet we doubt whether this principle can be extended to the length to which our author carries it. It is dubious whether a dog does not make as much urine proportionately, as a horse; yet the latter is exclusively herbivorous, and the former mainly carnivorous. It is well known that a horse in stable, fed on hay and corn, makes less water than when he is fed on "green-meat;" in other words, the quantity of urine is more or less proportioned to the quantity of water taken. This appears to us to be a general law in all classes of animals, a law which, in the absence of disturbing circumstances, seems consistent with reasoning and with experience.

We are far from convinced that animal food and strong drink have "a positive repressing influence on the secreting functions of the kidney." One's own knowledge of what happens at a dinner party leads to a contrary supposition. The chamber-pot is usually in requisition much in the ratio of the previous imbibition of good viands and good liquors. The well-worn corner of the outside of a pot-house offers evidence of the same sort. We have seen patients whose kidneys are always stimulated by ale—by coffee—by port wine.

So far as we can judge, stimulating liquors, like others, act upon the kidneys, unless the skin gets rid of the fluid in the shape of perspiration. The great difference seems to be, that if bland fluids are drunk, the urine is aqueous, and if acid and alcoholic fluids are taken it is more or less charged with the salts.

It cannot be expected that animal food should act much on the kidneys. Ex nihilo nihil fit: as that species of aliment has comparatively little water of composition, little water is obtained from it. It may furnish to the kidney an abundant supply of lithic acid, but aqueous urine in quantity is not to be looked for. In short, the kidney may be said to work on what it gets. If much water goes to it in any shape, that water, cæteris paribus, is discharged by it. If other matters, which the kidney is capable of separating go, the kidney, cæteris paribus, separates them. This appears to us a more simple expression of the facts, than the idea that animal food and strong drink positively repress the function of the kidney.

"That *dyspepsia,* or bad digestion," continues Dr. Willis, "in the ordinary acceptation of that term, is the most efficient cause of a lithic state of the urine, as has been so generally maintained, I cannot make up my mind to believe. My observation would lead me to say that there was a greater amount of weakness of stomach, characterized by heartburn and oppression after eating, in a single agricultural county in Scotland, than could be heard of over one half the surface of well-fed England. I have not found the urine of those who were habitual sufferers from dyspepsia, save on very rare and distant occasions, to deserve the epithet of lithic. I am, in particular, on habits of intimacy with two individuals, who in their persons present an epitome of all the symptoms of indigestion in their most painful and aggravated shapes, and the urine, in both cases, though always slightly acid when voided, is almost never high coloured, and seldom or never lets fall any deposite beyond the slight mucous cloud of health; when there is a slight precipitate in from twelve to twentyfour hours after the urine is passed, it consists of the lithate of ammonia of a pale colour, and in the amorphous state, *i. e.* the crystals are so small that they require a magnifying-glass to distinguish them.

That something else than simple indigestion is at work in inducing a lithic state of the urine and its consequences, calculous complaints, we have evidence in the fact, that whilst in the county of Norfolk one case of calculus occurs for every 21,000 of the inhabitants, and 10 and 12 operations for stone have for many successive years been annually performed into the Norwich Infirmary, the disease seems to be entirely unknown in the county of Hereford, no patient during more than 40 years having been received in the Hereford Infirmary labouring under stone, and no record of the operation of lithotomy ever having been performed within the bounds of the county being extant. In Devonshire, too, the disease of stone appears to be very rare." 66.

Two propositions are here mixed up. One proposition generally maintained is, that indigestion, not in a limited but in a comprehensive sense, is a cause of calculus—another proposition is, that locality, that is a combination of circumstances whose nature is at present imperfectly understood, is also a cause of stone. Each is to be proved or disproved by its appropriate evidence, and proving one (as they are not opposed in their terms) will not disprove the other. Indigestion is thought to be a cause of calculus, because it occurs on a large scale in those classes and at those periods of life on which and when the causes of indigestion operate. Another reason for the same conclusion is, that the symptoms of indigestion frequently precede or accompany calculus. If, in a particular county, where indigestion is not particularly rife, the complaint of calculus is so, that shews, of course, that the excess of calculus in that county is not due to indigestion. But it does not shew that it was not due to indigestion in the other cases. If, again, in another county, stone is scarce, though the average quantum of indigestion exists, that is presumptive evidence of the presence of some modifying circumstance there, but no decisive argument against the operation of indigestion in the other instances.

We freely admit that some, and those prevalent forms of indigestion, occur independently of lithic urine. Nothing, we conceive, can be more true than that. But indigestion is a wide word, and embraces the disturbed functions of many organs. It is difficult to say why one man after living freely gets pimples on his face, and lithic urine—while another is attacked with gastralgia and the blue devils—and a third with an attack of bile. Yet each of them is a phenomenon of indigestion, which usually in particular habits, assumes a particular predominating type. It is surely no argument against indigestion producing lithic urine in B, to urge that the indigestion of A was marked by a sick headache. As well might we tell A that it could not be his Welch-rabbit and ale that had disordered him, because the Norfolk chaw-bacons, who live on hard dumplings, cheese, and sour beer, are remarkable for calculus, and not for sick-headaches.

The fact is, that as digestion is an assemblage of many actions of as many organs, so indigestion is a comprehensive term which may embrace the disordered functions of all, or may signify the deranged condition of one. The kidney is as much an organ of digestion as the bowels. In many cases of dyspepsia, it is difficult to say why the organ that suffers should do so rather than the rest. In many others, we are able to determine the general predisposing, or particular exciting causes, that lead to the derangement of the organ in question. Thus, anxiety of mind and the fatigues of business principally affect the stomach. Sedentary occupations rather induce torpor of the liver or the bowels. Luxurious living breeds lithic acid and disturbance of the kidneys. One is as much indigestion in a wide and philosophical sense as the other. The constitutional states and the predispositions of the patients differ, the exciting causes vary, the organs chiefly implicated are not the same, but in each case the assimilating process is at fault, and the food is not converted in the normal manner into products or educts of digestion.

Dr. Willis follows out his views, and applies them not only to renal affections, but to other cases which by common consent have long been linked with dyspepsia—for example, gout. It would be injustice to him, as he urges the point strongly, and makes it a sort of cheval de bataille, not to allow him to speak for himself. As we do not coincide with him, this is the more necessary. Nothing can be more opposed to reason and justice than the modern system of reviewing. Cushioning, often garbling, or positively mis-stating the opinions of the writer, blazoning at unconscionable length his own, the critic of the present day retails his peculiar crotchets, or vents his private pique, under the garb of an impartial and conscientious censor of scientific truth. This infamous system ought to be put down. Reviewers ourselves, we cannot but feel, and we candidly own, that we have no right to pronounce dogmatic opinions. The reviewer is, after all, but an individual, often a very obscure one. Things seen through mists seem large; and it is the obscurity which shrouds the critic that lends him apparent magnitude and consequence. Could the public view him as he is, they would often find him but a sorry hack.

What right has such a man, what right has any man, to seize the chair of judgment, and try, absolve, or condemn unconditionally his peers or his superiors? Yet this *is* done without a blush, and the self-constituted tyranny is borne with. We have often thought, what a Falstaff's regiment the batch of periodical critics would make, could they only be paraded in the open day. What dirt, what meanness, what effrontery, what malignity would be marshalled! The fry of this day are the true descendants of the brood that smarted with the lash of Pope.

——— A feeble and a desperate pack,
With each a sickly brother at his back;
Sons of a day, just buoyant on the flood,
Then numbered with the puppies in the mud.
Ask ye their names? I could as soon disclose
The names of these blind puppies as of those;
An undistinguished brass their record bears—
"These are, ah no! these were the gazetteers."

But criticism certainly should not be a mere registering of an author's sentiments. Let criticism be as stringent as it may, but let the public be the judge. The reviewer, if he differs from his author, should expose the latter's sentiments as fully as his own. He may state his dissent, put his plea on the record, and leave the issue to the proper and the just tribunal of the public voice. Nothing, we repeat, can be more absurd or more iniquitous than that a man in a mask should affect to be the sole depositary of scientific knowledge, and ex cathedrâ denounce or extol the author whom he chooses to call before him.

We return to Dr. Willis, and having offered our reasons for quoting his sentiments, we quote them.

" Whilst I admit then to its fullest extent the influence of diet on the state of the urinary secretion, I am not prepared to say that this influence is engendered through any derangement of the digestive organs in particular, of the nature of that which we characterize under the name of dyspepsia or indigestion. It seems to me a subject of regret, that at the present day so many English writers should be found referring numerous and most dissimilar diseases to a single and similar source. The bias of one indisputably great man has communicated itself too widely in this direction. The digestive organs have had too much to answer for of late years; instead of being the beneficent instruments our forefathers regarded them, furnishing the frame with the pabulum needful to fit it for the discharge of high and onerous offices, they seem of late to have been looked upon as a kind of enemy in the camp, poisoning by their perverse operation the very sources of life, and throwing all the rest of the beautiful machinery of the body out of joint. But surely a indigestion is one thing, and gravel or stone another, and gout a third, and palsy a fourth, and tetanus a fifth, and chorea a sixth, and so on to the end of the chapter. Undoubtedly the digestive organs are simultaneously affected along with every form of general constitutional disturbance, as also with the particular derangements of each and every one of the separate systems of organs which make up the body; but because they suffer, and because their implication is forced in a peculiar manner on our notice, and because we mostly apply our remedies through the channels they afford us, they are not therefore the causes of these disorders. Let us take one in particular, among the host of diseases which it is almost universally the fashion to regard as depending on a disordered state of the digestive functions—*gout,* and ask, What is the actual state of matters preceding and connected with an attack of gout? Up almost to the hour of invasion the individual is generally in a state of unwonted good health; the stomach is doing its duty with alacrity; the body is replete with nourishment, the mind with energy. This individual retires to rest contented with himself, and with every one else, thankful to God, we shall presume, for all his blessings, and promising himself much farther cause for thankfulness in renewed and repeated enjoyment of these blessings. He goes to sleep and is lapped in Elysium. But by and by he is disturbed by uneasy sensations in the hand or foot, and is aroused to consciousness, wondering what can be amiss. The local pain increases in severity; sleep flies his pillow; he becomes restless and ill at ease; his pulse is full, his mouth parched, his skin hot and dry; and the remainder of the night, begun under such favourable auspices, is passed in misery, the patient tossing from side to side of his bed, and finding a resting place nowhere. The healthy man has become suddenly diseased; there is no whole spot about him, no function in his body that is not disordered, and the physician must be summoned. Now, in whatever the disease that has been developed consists, the stomach has certainly had no more part in its production than the heart or lungs, liver, or spleen, unless, indeed, as a casuist might maintain, it has been doing its duty too well. A diseased condition of the system, the effect, in so far as we know, of repletition, has manifested itself, accompanied and constituted by quickened circulation, suppressed secretions, disturbed digestion, local inflammation, and the other morbid symptoms, which we are accustomed to group together and call by the name of gout." 73.

It appears to us, that a question of this nature is not to be decided by any general declamation, but by an examination of the several propositions it includes. When Dr. Willis exclaims that "surely *a* indigestion is one thing, and gravel or stone another, and gout a third, and palsy a fourth, and tetanus a fifth, and chorea a sixth, and so on to the end of the chapter," he makes many affirmations, some of which may be correct and others not. Tetanus for instance may differ much more from indigestion than chorea, and chorea than gout. But the misfortune is that Dr. Willis does not define what he means by indigestion. We should rather suspect that he limits that term to one particular form of indigestion, to that which is characterized by gastralgia, palpable disturbance of the stomach and bowels, and hypochondriasis. If he does so limit the term, his proposition will be more correct than if he does not limit it. That is true enough, but limiting the term is arbitrary, opposed both to physiological and pathological reasoning, and inconsistent with correct generalization. We make these observations for the purpose of shewing how necessary it is, in announcing general propositions, to state explicitly the sense in which the terms are employed. It is impossible in the present case to determine how far we may coincide with Dr. Willis or differ from him.

But he descends to a special case, that of gout, and here we can more fairly meet him. He contends that prior to the attack of gout the individual is generally in unwonted good health, that the *stomach* has usually been doing its duty too well. Here again we perceive the bias of Dr. Willis to the notion that the stomach is *the* seat of indigestion. Surely the whole process of digestion is not performed by the stomach. That plays an important part, it is true, but the liver, pancreas, intestines, kidneys, play parts of some consequence also. Indigestion is a derangement of some part of the process of digestion, an imperfection or alteration of the functions of one or several of the organs which perform that process. This is the fair view of indigestion, this, we think, the view that is generally entertained. If, in ordinary indigestion, the stomach generally suffers most, that is only a matter of degree, a question of plus or minus.

But, taking our author on his own ground, we are far from satisfied that the attack of gout, is as a general rule preceded by unwonted good health. The gouty subject is generally known by his tendency to heart-burn (disordered secretion of the stomach)—to acid urine (disordered secretion of the kidney)—to flying pains—to the usual signs of plethora. Such is the result of our experience. We have not seen gout attack those in perfect good health. If by good health are meant corpulence, a ruddy face, a strong appetite, and good fellowship, undoubted signs of health in the popular creed, then gout does certainly supervene upon it. But, if we may trust our own experience, this apparent health, this actual plethora, covers derangement of the digestive organs characterized especially by the disposition to acidity in the primæ viæ and the urine.

The dictionary of Dr. Copland is acknowledged to be a valuable compilation. It expresses the sum of general experience, and may safely be referred to on a point like this. Let us hear what our excellent and learned friend has to say on the occasion:—

"*Of the premonitory Symptoms of the Paroxysm.*—Although the gouty paroxysm may attack suddenly a person apparently in good health, especially on the first occasion of its appearance, it is more frequently preceded by symptoms of disorder referrible chiefly to the digestive organs. I believe that, if the cases in which it is said to have appeared suddenly were investigated, it would be ascertained, that more or less disorder had existed for some days before the seizure, although not so as to have excited any concern in the mind of the patient. The most common symptoms of premonition are—flatulence, oppression after a meal, irregular appetite; heartburn, with acidity of stomach, sometimes with acid or acrid eructations; costiveness, irregularity, or, more rarely, an irritable state of the bowels; scanty, deep-coloured urine, becoming turbid or thick on cooling, or sometimes copious or pale urine; a sense of soreness, or occasionally of coldness, at the epigastric region; itching, or irritation of the skin; drowsiness, or frequent yawning, restless or unrefreshing sleep, more rarely nightmare; general lassitude and depression of spirits. In some persons, the symptoms of gastro-intestinal irritation are still more manifest, the tongue being loaded, red at its point and edges, the epigastrium tender, and the stomach oppressed after a meal. In many cases, increase of corpulency; scanty, thick urine; drowsiness, especially after eating, and a sense of general fulness and oppression, have preceded the paroxysm for a longer or shorter time, accompanied by several of the preceding symptoms. The appetite is frequently craving; and when indulged, is often followed by nausea, or vomiting of acrid matter, or by heartburn, flatulency, acrid eructations, &c. The premonitory symptoms vary in different persons, and depend much upon idiosyncrasy. Dr. MACKINTOSH justly remarks, that persons subject to gout are warned of a fit by some sensation or symptom peculiar to themselves individually: one feeling heat, pain, and dryness of the eyes; another, heat, redness, and swelling of the nose; a third, an unusual craving for some particular kind of food, or some peculiar feeling at the stomach, &c. Palpitations or internal flutterings; severe cough, with mucous expectoration; irritability of the bladder, the urine being loaded with mucus; a discharge from the urethra, with scalding, or difficulty in passing the water; unusual lassitude, and inaptitude for mental exertion; peevishness, irritability of temper; depression of spirits, more rarely an unusual hilarity; and various other symptoms, severally precede the paroxysm in different cases."*

We apprehend that the representations of Dr. Copland and ourselves will be found more conformable with general experience than those of Dr. Willis.

We have entered fully upon this question and urged our objections to the views of Dr. Willis. Those views may be said to be peculiar to himself—our objections represent the opinions commonly received. We state them with candour and without reserve, and we are sure that Dr. Willis will admit the propriety of fair and free discussion. By such discussion we may lead him either to modify his opinions, or to confirm them by stronger proofs. As truth is our object, we shall be equally satisfied in either case.

So far as we can see, the following facts would seem to be tolerably certain:—

1. *That*, particular kinds of aliment, liquid and solid, lead to the lithic diathesis.

2. *That* the kidney being one of the organs employed in the apparatus and process of digestion, disturbance of its function is, pro tanto, disturbance of the function of digestion.

3. *That*, this disturbance of the function of the kidney, is often, if not generally, preceded or accompanied by disturbance in the other organs of digestion, especially the stomach.

4. *That*, the lithic diathesis is usually attended with acidity in the primæ viæ, and often with the phenomena that characterize the gouty habit.

---

* Dictionary of Practical Medicine. Part IV.

We have not touched on an interesting and an important subject, the condition of the fluids in this case. Who that considers the phenomena of plethora, of gout, of lithiasis, can doubt that disorder of the digestive organs is but a part, the initiative and the conclusive, in the circle of vicious actions. Food too highly charged with particular ingredients is introduced into the primæ viæ. The digestive organs form from that food a circulating fluid which must be loaded with the same ingredients. By and bye occurs disorder of one of the eliminating organs, the kidney, or of another, the skin: or of the whole system. Must we not suspect that the view which is restricted to the particular disturbance of skin, or kidney, is a narrow and deceptive one? Must we not conclude, so far as general reasoning can lead us to conclude any thing, *that* a general morbid condition exists, of which these detached disturbances are only parts, and *that* this morbid condition must be sought and found in the blood? With the progress of organic chemistry, we may expect an extension of our present limited pathological views.

Pursuing the causes that give rise to lithic urine, Dr. Willis enumerates—sedentary habits, especially in those who have previously been accustomed to the contrary—injuries to the kidney itself, a circumstance especially insisted on by the late Mr. Earle—the diminished temperature of the body which attends old age.

This latter cause was first pointed out by M. Magendie. By actual experiment he found the temperature of the axilla in men above 60 to be seldom above 36° C. or 96°.5 F. nearly. The urine projected upon the thermometer he found no higher than 30° C., about 86° F., or ten degrees of the latter scale under the temperature proper to the vigour of life. Ten degrees of temperature must certainly have some influence on the solvent powers of the urinous menstruum. These experiments of M. Magendie have been confirmed by others reported by M. Chevallier, who remarked that the temperature of the urine in children was low, that of adults higher, and that of the aged again low. The urine of a healthy man aged 55 was no higher than 33° C. or 91°.5 F.

Another cause of the lithic diathesis is that inherent peculiarity of constitution which is universally admitted to be similar to that which is disposed to gout.

And finally cold, particularly when applied in conjunction with great and sudden vicissitudes of temperature, appears to be an efficient agent in the production of the malady. The county of Norfolk, observes our author, presents generally an exposed and open surface; the alternations of temperature are great and sudden, and there is no district in England over which the east wind, the dread of the invalid and person of susceptible nervous system, sweeps more constantly or more searchingly than over Norfolk. Suppressions of the cutaneous exhalation, combined with the kind of food generally used, are in all likelihood the potent cause of those derangements of the urinary secretion, which lead to the superabundant elaboration, and deposite of some of its suspended, or dissolved but readily concrescible ingredients, within the body in that division of this country.

Free action of the skin seems incompatible with the lithic diathesis. At all events it tends very powerfully to moderate it.

"The immediate cause of the precipitation of the lithic acid, especially when this takes place within the body, has been variously accounted for. Those who maintain that the acid exists in combination with ammonia, by which it is rendered more soluble, explain the occurrence readily through the agency of the free acid, which is always present in lithic urine. This free acid, say they, dispossesses the lithic acid of the base with which it is combined, and causes it to precipitate. The soundness of this explanation, however, is very questionable. First, the lithic acid separated from an alkaline base in this manner, is thrown down in a pulverulent or amorphous form, not in distinct crystals, as we perceive it to be, and when its consequences are alone to be apprehended. Then, there are great doubts in regard to the nature of the acid which can be spared to have such an effect. Dr. Prout supposed the muriatic acid to be the agent; but this acid has never been shown to exist in the urine, save already saturated with bases—ammonia, potash, and soda. The phosphoric acid has enough to do in holding the lime with which it is associated in solution. The lactic acid, the free acid of the urine, according to Berzelius, has been shown by Wetzlar to be the least efficient of all the acids in causing the precipitation of lithic acid from its state of solution in the urine. Finally, it is all but certain that the explanation of the precipitation of lithic acid from the urine by the agency of any free acid is erroneous. If the lithate of ammonia often occurs in the urine, it is no less certain that the free and uncombined lithic acid occurs there too. It does not seem necessary to the writer to search so anxiously for a chemical cause of the phenomenon in question. It is enough to say that in certain states of the system, under the influence of peculiar disturbed conditions of the vitality of the kidney, a very insoluble principle is separated in large quantity by this organ, the natural consequence of which is, that its particles, at the moment of their formation, are attracted by their inherent electivity, and concrete into the crystalline forms that belong to them: the precipitation is a *labes vitalis*, an error of vitality in the secreting organ, the kidney." 79.

The tendency of the lithic diathesis need not be insisted on. If unchecked, it leads to lithic sand and calculi.

*Treatment.*—This is well understood. The causes, whatever they may have been, which occasioned the complaint ought, if possible, to be removed. Sedentary habits, high diet, exposure to cold, and light clothing, must necessarily be proscribed.

If the attack is too severe to permit confidence to be placed in regimen alone, cupping on the loins, smart purging, salines, and antiphlogistic diet, should be had recourse to.

" In the first period of a plan of treatment of this kind, it is not uncommon to see a considerable quantity of crystallized lithic acid discharged from the kidney, an event which has been observed to follow with considerable regularity upon the use of some of the ordinary diuretics, such as the turpentines. Whether this discharge of lithic acid is to be regarded as critical, *i. e.* as a kind of materies morbi eliminated from the system by the kidney, or as a simple expulsion from the pelves of the kidneys of a quantity of concrete foreign matter, has been made subject of question among pathologists. In some cases there seems little reason to doubt of the critical nature of the discharge; in others, it is obviously the effect of the mechanical and chemical action of the diluents that are so uniformly prescribed when red sand has been observed to be passed along with the urine. Should any thing like uneasinesss continue in the region of the kidneys, after the measures which have been specified have been enforced for some days, it may be well to try the effect of one or two doses of turpentine. If they have the effect of bringing away a quantity of sabulous matter, the relief experienced by the patient is always great; and the good effects of the medicine may be secured by the subsequent use of gentle aperients, diluents, regulated diet, &c. I do not know that serious mischief has ever actually happened from the attempts which are sometimes made, at all hazards, to induce such an elimination of lithic acid from the kidney, by combining the terebinthinate diuretic medicine with muriatic acid and opium. It seems to me just possible that a course of this kind might lay the foundation of a renal calculus, with all its immediate and prospective miseries. I can hardly conceive a case, therefore, in which I should be inclined to adopt it; certainly not one in which I should persevere with it longer than a day or two. We have better than purely empirical grounds to go upon in the treatment of snch cases." 83.

If there has been injury of the kidney, or reason to believe that it has been in any way put severely to the test, repeated leeches or cupping on the loins, and even the insertion of a seton, will be requisite. The benefits of counter-irritation can hardly be over-rated.

Mercurial alteratives and gentle aperients are of great utility.

" But there is another, and as it might be truly called, specific plan of treatment that ought not to be neglected in the lithic diathesis. This consists in the exhibition of the alkaline bicarbonates in a state of large dilution, or in a course of one of the mineral waters which contains a bicarbonated alkali among its saline ingredients. Sulphur is hardly if at all more potent against scabies, than the alkaline bicarbonates are influential against a disposition in the urine to deposit lithic acid. Taken at proper times, and dissolved in plenty of water, these salts are perfectly innocuous, and may be continued for months, and even years, not only without injury, but in many cases with great benefit to the general health. From half a dram to a dram of the bicarbonate of potash or soda (the soda is probably the more german to the system) dissolved in from three quarters of a pint to a pint of thin mucilaginous fluid, should be taken two or three times a day according to circumstances, at periods as remote as possible from those at which meals are taken. Immediately before or immediately after a full meal, a large dose of either of these salts will not fail to interfere with the important process of digestion. Schwann* found that they destroyed the pecnliar faculty of the Pepsin or the digestive element. Administered at the distance of two hours before a meal, they are abstracted from the stomach and carried off into the torrent of the circulation long before any food is taken, and the digestive process goes on unimpeded by their presence in the blood. If the patient's circumstances permit him to leave his ordinary place of residence or business, and betake himself to a watering-place for a few months, he can do this to greater advantage in no circumstances than when he is labouring under a lithic state of the urine. Our own country is deficient in mineral waters that are peculiarly adapted to such a case. But on the Continent they abound. At the head of the whole may be placed the waters of Vichy, in the centre of France. No mineral water known contains so much bicarbonate of soda as the hot springs at Vichy; an ordinary tumblerful holds in solution nearly eighteen grains of this salt; and two or three glasses taken at intervals in the course of the day, are often sufficient to maintain the urine in a neutral or alkaline state, nay, the mere soaking in a warm bath of the water is sufficient to produce the same effect.† The waters of Mont d'Or, Carlsbad, Selters, Pyrmont, Obersalzbrunn, &c. have the same effect in different degrees; they each prove beneficial in such cases at all events. Where removal is inexpedient or impossible, we can supply the place of these natural alkaline waters to a considerable extent, by prescribing the bicarbonate of soda or potash in a proper quantity of thin barley-water, or in simple water

* " Ueber das Wesen des Verdauungs-Processes, Berl.; also Mueller's Physiology Transl. by Baly, p. 545."

† " D'Arcet, in Ann. de Chimie, 1826."

impregnated with carbonic acid gas.* Fifteen or twenty grains of the bicarbonate of soda, dissolved in two ounces of water, having a half-pint bottle of our ordinary soda-water (water charged with carbonic acid gas) poured over it, makes an artificial mineral water that is extremely pleasant to the palate, and is taken freely by patients." 86.

It is rather singular that Dr. Willis makes no mention of colchicum in the treatment of the lithic diathesis. Yet perhaps there are no remedies, not even excepting the alkaline bicarbonates, more useful than it is. The combination of colchicum with the liquor potassæ is extremely valuable. There are several other medicines and combinations of medicines, of service in this malady, to which Dr. Willis fails to allude. We cannot consider his remarks on its treatment comprehensive.

We conclude the examination of the lithic diathesis, by quoting the directions for the chemical examination of lithic acid and its deposits.

A known quantity, say 1000 grains of the urine, after it has stood for several hours, is to be poured upon a filter of fine bibulous paper, and the fluid separated from the solid parts. Another portion of the urine may be tested for any additional lithic acid it may contain, by having a few drops of muriatic acid added to it. The precipitation of the lithic acid may always be immediately secured by drying well and warming gently the glass vessel into which the urine about to be examined is poured, or by rubbing the inner surface of the vessel containing the urine after a few drops of acid have been added to it, with a rod of wood.* The colour of the deposites is best ascertained whilst they are still wet upon the surface of the filter. The fluid portion is to be treated in the manner already recommended at the end of the first chapter, by which the amount and relative proportions of the principal classes of ingredients will be ascertained.

To examine the nature of the deposites, let a known quantity of any of them be boiled with distilled water, by which any alkaline lithates will be dissolved. The solution being evaporated to dryness, a portion of the extract should have a few drops of nitric acid poured upon it; this will occasion the solution with effervescence of the lithic acid and the lithates. The nitric acid solution being dried by the application of gentle heat, the pink colour characteristic of the purpuric acid (formed by the action of the nitric on the lithic acid) will be evolved, and this tint will immediately pass into a bright purple on the addition of a little caustic ammonia. By this means we detect lithic acid. Another portion of the extract being mixed with some caustic lime, if it contains ammonia, this will be discovered by the smell, or by approaching a rod dipped in muriatic acid to the mixture. A third portion of the extract must be burned over the flame of a lamp in a platinum spoon, and the residue tested for alkaline re-action. The nature of the alkali found, whether potash, soda, or lime, is discovered by urging the residue for

---

* The following is the Receipt in the French Codex for the artificial Vichy water: Carb. of soda, 1 dram, 56 grains; chloride of calcium, 11 grains; chloride of sodium, one-third of a grain; sulph. of soda, 6 grains; sulph. of magnesia, 3 grains; sulph. of iron, one-third of a grain; water, deprived of air, 20 oz., carb. acid gas, 3 volumes and a half.

* Wetzlar, l. c. who found he could obtain a deposite of lithic acid by this means, in many cases where the simple addition of an acid failed to produce it.

a moment with the flame of the blow-pipe. If it be soda the outer flame will be coloured yellow. Potash and lime are distinguished by the solubility of the former in distilled water; or should the lime exist in the caustic state, when it forms an oxide of the base and becomes soluble, by passing a current of carbonic acid gas through the mixed solution, or adding a few drops of a solution of oxalic acid, it will immediately fall. The presence of the earthy salts—the ammonia-phosphate of magnesia, and the phosphate of lime—is known by the insolubility of the deposit in boiling distilled water, and by its ready solubility in dilute acids, from which the earthy bases are precipitated on the addition of an alkali. The relative proportion of the differently soluble lithates and the insoluble phosphates may be ascertained by digesting a known weight of the mixed precipitate in a sufficient quantity of attenuated caustic potash, or soda, which takes up the lithates, and leaves the earthy phosphates behind.

A recent great improvement in the analytic investigation of urinary deposites, introduced in the service of M. Rayer, and well described by M. Vigla, is the examination of these with the microscope.

We pass to the second, the remaining section of this Chapter, on—

*Sedimentary Urine in which the Deposite consists of the Earthy and Earthy-Alkaline Phosphates.*

The earthy phosphates are only held in solution by the excess of acid with which they are combined, or in consequence of the presence of some other free acid in the urine, which can in many cases be demonstrated to be the carbonic. The *neutral phosphate of lime** is a salt to all intents and purposes insoluble. *Phosphate of magnesia*, another urinous salt, is not very soluble, requiring at least fifty times its weight of water to take it up. *Phosphate of ammonia*, indeed, is soluble enough by itself, but in combination with the phosphate of magnesia, as it always exists in urine, it becomes exceedingly insoluble. A slight excess of phosphoric acid, and, as it would appear, the presence of carbonic acid in some cases, put an end to the insoluble character of these salts; as super-phosphates or carbo-phosphates they become readily soluble, and are safely carried out of the system.

The phosphatic sediments may occur either in the crystallized or amorphous state. In the former case the crystals are white and glistening, and their formation is frequently accompanied with the precipitation at the same time of a certain quantity of the amorphous lithic sediment. The lithic deposits are consistent, we need scarcely say, with apparently robust health, and plethora. The phosphatic diathesis is well known to be just the contrary.

At first, the phosphatic state of the urine is generally found to alternate with that in which the cream-coloured or pale-tawny amorphous lithic sediment occurs. By and bye the lithic deposites are observed less frequently, and, when the constitution is quite broken, not at all.

The urine is always pale-coloured when passed—secreted in excess—sometimes weakly acid when first voided, or neutral; as the disease increases, it never fails to become alkaline. It passes very rapidly into putrefaction.

---

* Dr. Henry reckons the quantity at as much as half a grain to each ounce of healthy urine. Elem. of Chem, vol. ii.

"Its specific gravity varies greatly according to the period of the day when it is examined; I have found it as low as 1,004 in the early part of the day, and as high as 1,033 about eleven o'clock at night, the time when I have always found the urine to possess the highest density." 92.

Crystals of the phosphates are rarely deposited within the body. Yet every now and then we find a stone with a phosphatic nucleus. The outer laminæ of stones are very commonly phosphatic. Crystals of the ammonio-magnesian phosphate constitute white sand.

The amorphous phosphatic sediments are much more common. Phosphatic urine is either a consequence of grave constitutional disturbance, or of great irritation or structural disease of some of the urinary organs. It occurs among the exhausted and unhealthy, and is a constant sequence of other diseases of the urinary organs. The irritability of system and constitutional disturbance that preceded or accompanied the diathesis are aggravated as it proceeds.

"The urine in this state of affairs is not only disordered in quality; it is often secreted in excessive quantity, sometimes to the extent that has been noticed under the head of hydruria. It is always pale-coloured, and if transparent when first voided, which, indeed, is rarely the case, it grows turbid as it cools, and lets fall an abundant precipitate of a white impalpable powder, consisting of the combined phosphates of ammonia and magnesia, and phosphate of lime. If brought to the boiling point, a flocculent deposite takes place, which has often been mistaken for albumen, but which consists principally of the phosphate of lime, as is immediately made manifest by the addition of a few drops of nitric acid, which re-dissolve the precipitate and restore or give complete transparency to the liquid. On standing, it hardly ever becomes perfectly transparent. Even within a few hours it is covered on the surface with a delicate pellicle, which is often iridescent, and on the lower surface of which well-shaped crystals of the triple phosphate of magnesia and ammonia are rapidly formed and sink through the fluid when it is disturbed, or when they become so heavy as to break away from the film. The urine anon becomes ammoniacal, and then the deposite goes on still more rapidly than before. Sometimes it is alkaline even at the moment it is voided; in any case, and when the temperature is between 50° and 70° F. it is not long before it begins to putrefy, and to evolve ammonia in abundance." 95.

The facts here stated have been pointed out by two or three observers, and have been known for some time to most who are conversant with urinary diseases.

We observe nothing particular in regard to treatment, with the exception of a very heterodox opinion on the subject of bread. "Bread," says our author, "that is six or eight hours out of the oven,—a French roll within one hour after it has been drawn, is, according to my experience, far more easily digested, and also much more nutritious than that which is two days old." We doubt whether many converts will be made to this doctrine. The common experience of us all would seem to have decided against new bread. Our author considers the lighter French and Rhenish wines objectionable, as having a decided tendency towards the kidneys. We have certainly seen them agree very well in cases of alkaline urine, and we have also seen them disagree. Dr. Willis recommends a glass or two of dry old sherry, that wine which, of all others, ought to be under deep obligations to the faculty, for it is prescribed on almost all occasions. Dr. Willis speaks particularly in praise of the fine Spanish wine called amontillado.

Dr. Willis remarks—

"The present very prevalent custom adopted in England from our continental neighbours of using *lavements,* as a means of evacuating the bowels, ought not to be so much encouraged as it is. Nature sent us into the world without a glyster apparatus; and if the lower bowel be for a while hindered of its proper office, it will cease from its duty of expelling the matter accumulated within its cavity." 99.

We cannot say that this mode of reasoning is convincing. Nature sent us into the world without many other things besides glysters, which yet are acknowledged to be useful. No doubt it is absurd for an individual whose bowels act well spontaneously to resort to glysters. But if aperients are requisite, and a glyster will answer, surely it is a more innocent remedy than a stimulating purgative substance. But this does not seem to us the right method of considering or determining the matter. The bowels are torpid. To remedy that state we must ascertain on what it depends. It may be on deficient action of the liver, of the small intestines, of the large. Glysters are of little service to those who labour under torpid livers—of great advantage to those whose large intestines allow fæces to collect and inspissate in them, without any adequate efforts to expel them. This is the practical view we should take. Some persons derive great advantage from glysters, others find little benefit from their employment.

On the whole, the methodus medendi advised by Dr. Willis is a repetition of what has been so forcibly insisted on by Dr. Prout.

We may sum up the principles and items of treatment at no inordinate length—light, nutritious diet—" amontillado"—gentle aperients, to the exclusion of mercury and of saline medicines—opiates to quiet the urinary apparatus—then, " some one or other of the articles which experience has shown to possess a kind of specific influence in allaying irritability of the urinary organs, particularly the uva ursi, pareira brava, and alchemilla arvensis. An extract of the uva-ursi, combined with extract of hyoscyamus or opium, according to circumstances, has been strongly recommended by Dr. Prout. The Ferrum sesquichloridum of the last London Pharmacopœia, or its tincture, is an excellent chalybeate; and when the phosphatic diathesis can be obviously referred to affections of the urethra, prostate, or bladder, the ferrum ammonia-chloridum, or its tincture, may be substituted, and will be found an invaluable medicine"—and, lastly, country air, and the encouragement of cheerfulness and hope—such is the category of remedial measures.

We would merely offer one or two observations. We have seen the phosphatic diathesis, certainly not in a marked degree, ensue from high living. A gentleman was in a situation in which he had a good deal of corporeal exertion, and lived moderately, taking animal food only at his dinner. A rather sudden change occurred. He took animal food twice or thrice daily—drank more wine—took less regular exercise. His urine now became alkaline. A repetition of the same circumstances has always subsequently produced alkalescence. Moderate living, active exercise, and only a glass or two of wine, render the urine acid. It is now generally disposed to be neutral, yet if this gentleman walks eight or ten miles daily it grows even high-coloured. In this case the circumstances that usually occasion lithic urine give rise to the phosphatic. The gentleman is not strong, and has used a fair share of mental exertion.

We have seen in some cases, certainly not always, much benefit from sarsaparilla. Occasionally it disorders the stomach. The infusion with infusion of rhubarb has agreed when all other forms have failed.

If, on the subject of the lithic and phosphatic diathesis, little is added by our author to what had been previously given to the world by Dr. Prout, it is because it would be impossible to add much. The progress of chemistry may correct some errors on the part of that gentleman, fill up some deficiencies, modify some opinions. But his work will still remain a model of the philosophical application of chemistry to medicine, and a standard work on the subject that it treats of.

We must now pause in our examination of the Treatise of Dr. Willis. That gentleman is a man of extensive information and of judgment. The length to which we have already proceeded is an earnest of our estimate of his own talents and the value of his work. To expect novelty on such a subject and after what has been written on it by men like Marcet, Prout, Brodie, &c., would be unjust. But Dr. Willis has thrown together what has at different times and by different men been given to the public, and has added the reflections of an observant and cultivated mind.

We shall pursue in our next number the analysis of this volume, and we shall present an epitome of the actual state of information on diseases of the urinary organs—an epitome which, we conceive, will be far from useless to the practical portion of our readers.

We have not hitherto availed ourselves of the work of M. Rayer. We received it rather late, and have deferred until the succeeding article,* a particular reference to its contents.

The work itself is extremely valuable. The plates are highly finished, the scheme comprehensive, the details minute.

M. Rayer is one of the few French authors who have any thing like a pretension to acquaintance with English medical literature. The consequence is, what might have been anticipated, that M. Rayer's works are much more practical than those of most of his countrymen. From his pen we have not mere mysticism, generalities, abstractions, and transcendentalism, but that substantial information adapted to the common purposes of life, and constituting useful knowledge.

---

* In our next Number.

